# Liquid Nitrous Oxide as an Anæsthetic

**Published:** 1872-02-01

**Authors:** Edmund Andrews

**Affiliations:** Professor of Surgery in Chicago Medical College; No. 6 Sixteenth St., Chicago


### LIQUID NITROUS OXIDE AS AN ANÆSTHETIC.

BY EDMUND ANDREWS, M. D.

*Professor of Surgery in Chicago Medical College.*

Many months ago I made experiments on the use of nitrous oxide pure, and nitrous oxide mixed with oxygen, with the view of adapting them to the purposes of anæsthesia in general surgery. These experiments, which were published in THE EXAMINER, showed that there were several difficulties in the way of using, for prolonged operations, the ordinary gas of the dentists—one of which was that the gas never makes the least approach to purity, containing always from 10 to 25 per cent. of free nitrogen and oxygen. As it is almost impossible to separate the free nitrogen without an apparatus for reducing the gas to a liquid form, I suspended the experiments until this deficiency could be supplied.

Three weeks ago I received from Johnston Brothers' Dental Depot, 812 Broadway, New York, an apparatus and a supply of liquid nitrous oxide equal to 100 gallons of gas. It is well-known that under a pressure of about 750 pounds to the square inch nitrous oxide condenses to a liquid, while the free nitrogen and oxygen, contained as impurities in it, remain in the gaseous form in the top of the receiver, hence the condensation serves as a means of separating the oxide from the other gases. The liquid was contained in a strong iron flask about four inches wide and twelve inches long. When it is desired to use the anæsthetic a faucet is turned slightly, allowing the gas to escape into a rubber tube leading to a small sack, and thence to an ordinary inhaler, so contrived, with valves, that the inspirations are drawn from the sack and the expirations pass into the open air. When not in use the whole is packed in a neat case about five inches square and fourteen inches long, with a handle on one side for carrying it, and weighs, perhaps, twelve pounds. The contents of one flask will last long enough for about three surgical operations of moderate length. The intention is to have the flasks kept ready filled at drug stores and dental depots, where the surgeon can return his empty flasks and procure filled ones.

The apparatus being new to myself and assistants, it is probable that we did not obtain the best possible results from it, but the following cases illustrate the effects:

*Case* 1. Five fingers to be amputated for frost bite, followed by mortification. The patient came under the influence slowly and with difficulty. At length his consciousness seemed to be gone, and his countenance presented the blue asphyxiated look characteristic of full anæsthesia by nitrous oxide, nevertheless he writhed and struggled during a large part of the operation, and afterwards complained that he felt every cut. The result was decidedly unsatisfactory.

*Case* 2. This was mortification of a single finger requiring amputation. The patient was given the gas more freely, by turning on a more copious stream from the flask. He was anæsthetized in about forty seconds, and went through the operation without any consciousness of pain.

*Case* 3. This was an operation upon varicose veins in the leg. The patient went under the influence of the anæsthetic in 35 seconds, obtaining a complete insensibility. In this state I injected the veins of the leg in about ten places with tincture of iron, without causing any pain.

*Case* 4. The inhalation commenced, but the supply of gas gave out, and the anæsthesia was completed with ether.

It is stated that the operation of ovariotomy has been performed under the successful influence of this anæsthetic in New York.

The present state of knowledge on the subject, derived largely from my own investigations, may be summed up in the following propositions:

1. Nitrous oxide is the safest anæsthetic known. In a laborious collection of statistics which I made, embracing over 200,000 cases, I showed that the mortality of these articles was nearly as follows:

Chloroform, one death in 2,723 cases; sulphuric ether, one death in 23,204 cases; mixed chloroform and ether, one death in 5,588; bichloride of methylene, one death in about 7,000; nitrous oxide, no death in 75,000. (Chicago Medical Examiner, 1870.)

2. Nitrous oxide anæsthetizes the patient in about forty seconds, while the other articles require from five to twenty minutes. The patient also awakes quickly, and does not suffer from nausea afterwards.

3. The liquefaction of the gas renders it portable, so that it can now be easily carried to patients' houses.

4. The expense, after the manufacture and sale are well established, will probably be less than that of ether.

5. Nitrous oxide always produces a purplish flush of the face, indicating partial asphyxia, though without any sense of dyspnœa, hence it cannot, in prolonged operations, be given continuously, but must be alternated with short intervals of breathing atmospheric air, renewing the inhalation of the gas as often as the duskiness of the countenance subsides.

6. It is not certain yet that all patients can be kept steadily asleep in this manner. Some of them seem to arouse after the first short sleep, and cannot be again fully quieted, even if the gas be pushed to the production of a decided flush of asphyxia in the countenance. Perhaps further experiments will remove this difficulty.

7. Nitrous oxide, in the gaseous form, may be mixed with one-fourth its bulk of free oxygen, and then does not asphyxiate. In my experiments on this subject I kept one patient nine minutes breathing the mixture, but the anæsthesia was less profound than was desirable.

8. If the patient be kept absolutely on pure nitrous oxide alone, he will die by asphyxia in spite of the general safety of the article, as it is incapable of oxidating the blood. The assistant administering it must pay attention to this matter, and give atmospheric air as required. I placed a rat in nitrous oxide gas from a dentist's gasometer, and the animal died in ten minutes, while another rat placed in a jar of the gas mixed with pure oxygen, remained in it half an hour and was taken out alive.

9. Although it is not yet certain that the nitrous oxide can be made generally available for long operations, it is already for all short ones the most convenient, safe and pleasant anæsthetic known.

No. 6 Sixteenth St., CHICAGO, Jan. 10, 1872.

THE REMOVAL OF AN INVERTED WOMB.—Dr. Thomas Hay, of York, Pa. (*Med. and Surg. Reporter*), lately removed an inverted uterus with an intramural fibrous tumor of the fundus. Four weeks afterward the patient was up and about her room, and the operation bade fair to be a perfect success.—*Ibid.*

